# From helplessness to controllability: toward a neuroscience of resilience

**DOI:** 10.3389/fpsyt.2023.1170417

**Published:** 2023-05-03

**Authors:** Michael V. Baratta, Martin E. P. Seligman, Steven F. Maier

**Affiliations:** ^1^Department of Psychology and Neuroscience, University of Colorado Boulder, Boulder, CO, United States; ^2^Positive Psychology Center, University of Pennsylvania, Philadelphia, PA, United States

**Keywords:** learned helplessness, stressor controllability, medial prefrontal cortex, dorsal raphe nucleus, depression

## Abstract

“Learned helplessness” refers to debilitating outcomes, such as passivity and increased fear, that follow an uncontrollable adverse event, but do not when that event is controllable. The original explanation argued that when events are uncontrollable the animal learns that outcomes are independent of its behavior, and that this is the active ingredient in producing the effects. Controllable adverse events, in contrast, fail to produce these outcomes because they lack the active uncontrollability element. Recent work on the neural basis of helplessness, however, takes the opposite view. Prolonged exposure to aversive stimulation *per se* produces the debilitation by potent activation of serotonergic neurons in the brainstem dorsal raphe nucleus. Debilitation is prevented with an instrumental controlling response, which activates prefrontal circuitry detecting control and subsequently blunting the dorsal raphe nucleus response. Furthermore, learning control alters the prefrontal response to future adverse events, thereby preventing debilitation and producing long-term resiliency. The general implications of these neuroscience findings may apply to psychological therapy and prevention, in particular by suggesting the importance of cognitions and control, rather than habits of control.

## 1. Introduction

“Learned helplessness” was first used by Overmier and Seligman (1) and “controllability” was first coined and then explicitly manipulated by Seligman and Maier (2). That work has had some influence on the field (3–5), so a review of how this field developed and the reasons for the directions that we took is in order. After this review we will describe more recent work exploring the neural mechanisms that underlie stressor controllability as well as how this clarifies several open, puzzling psychological issues. Finally, we will speculate about the clinical and cognitive implications of the neuroscientific findings.

## 2. Early thinking

Maier and Seligman began this work while graduate students in the laboratory of Richard L. Solomon at the University of Pennsylvania. By accident two senior graduate students in the lab, Russell Leaf and J. Bruce Overmier, had found that first exposing dogs to 64 trials of aversive Pavlovian conditioning in which a tone CS (conditioned stimulus) was paired with a footshock UCS (unconditioned stimulus) in one environment led to later failure to learn to escape grid-shock in a shuttlebox (6). This phenomenon was quite dramatic as the subjects merely had to jump over a small hurdle to the other side to terminate shock, something learned very easily by control subjects: yet prior Pavlovian conditioning led to complete failure to learn in most subjects. Seligman and Maier set out to determine its causes. To begin analyzing the phenomenon Overmier and Seligman (1) eliminated the tone and provided only the foot-shocks in the first phase. In phase 1, as defines “Pavlovian Conditioning,” the UCS, the footshocks were uncontrollable—that is, inescapable and unavoidable. Nothing the subject does in Pavlovian conditioning can influence the UCS, otherwise it would not be Pavlovian conditioning. Eliminating the tone did not change the failure to learn to escape in the shuttlebox later. Overmier and Seligman (1) speculated that perhaps “the source of the interference is a learned ‘helplessness.’” Learned helplessness might well result from receiving aversive stimuli in a situation (such as Pavlovian conditioning) in which all instrumental responses are of no avail in eliminating the trauma.

Starting from this nascent idea, Seligman and Maier (2) introduced the notion of “control.” They reasoned that organisms could learn that their responses were “of no avail,” and such learning would undermine trying in subsequent situations, and they tested this hypothesis with the “triadic design.” Thus, one group (Escapable—ES) could end each of the shocks by pressing a panel. That is, the subjects had an escape response and so there was an instrumental response that was of avail. For a second group (Inescapable—IS) each member was “yoked” to one of the animals in the escape group. Each shock began at the same time as for the escapable subject and ended whenever its escape partner pressed the panel. For IS, shocks were inescapable. Thus, the two groups were exposed to physically identical aversive events, but one had what Seligman and Maier called “control” over duration of the aversive event. A third group did not receive any shocks. The Inescapable group later failed to learn to escape in the shuttlebox, while the Escapable group learned well, as did the non-shocked controls. That is, prior shock *per se* did not produce interference, but only uncontrollable shock did.

In a further experiment, Seligman and Maier reasoned that a first experience with escapable shock should interfere with the organism’s later learning that it had no control: so later inescapable shock should now not produce interference with shuttlebox escape learning. This was the case, and we called this protection “immunization.”

From here the “learned helplessness hypothesis” was developed (7). It should be noted that in 1967 very little was known about stressors, and “stress” itself was not a frequent topic within psychology. PubMed shows only 158 hits for the term “psychological stress” in 1967, and the huge majority did not measure behavior, but rather blood pressure, hormonal responses, and so forth. Contrast this with the over 14,000 hits in 2022. The only known behavioral effect of exposure to inescapable shock was ours, and so we developed an explanation of why it occurs. Maier and Seligman wondered why inescapable shock produced interference with escape, but they did not wonder why escapable shock failed to produce interference since widespread behavioral effects of aversive events were not known. Indeed, “stress” did not appear at all in any of the early papers, and they viewed the interference as strictly a learning phenomenon. They did not think the fact that inescapable shock is stressful was of importance here, and we predicted, for example, that inescapable food would similarly interfere with later learning responses to obtain food.

Seligman and Maier next set out to explain what the organism learned during exposure to inescapable shock and how this learning might interfere with the subsequent acquisition of escape in a different environment. It was reasonable to focus on what was learned as causal since the groups that received escapable and yoked inescapable shocks were exposed to physically identical stimuli, and so the difference between them had to be psychological. Moreover, it was reasonable to focus on what the subjects in the inescapable group learned rather than what the escape subjects learned since the difference from non-shocked controls, occurred only in the inescapable group.

What was learned? According to the behavioristic thinking dominant in 1967, responses, but not cognitions, were learned, and the **contiguous** pairing in time of a response and a reinforcer (here shock termination) strengthened responses, while non-reinforcement following responses weakened responses. Instead, Maier and Seligman argued (8) that organisms learn cognitively about the **contingency** between responses and outcomes. They have come to prefer “act” to “response,” since it has never been clear with voluntary action what the “response” is responding to. **Contiguity** is determined by a single parameter—the conditional probability of the outcome given the act. **Contingency** is instead defined by two parameters, the conditional probability of the outcome given the act and the conditional probability of the outcome in the absence of the act. When the two probabilities are unequal there is a contingency, and the organism can increase the likelihood of the outcome by either doing or withholding the act. When these two probabilities are equal, however, action, by definition, does not affect the occurrence of the outcome. Thus, when the two probabilities are *unequal* the organism can control the outcome and when they are *equal* for all actions it cannot. When shock is inescapable the two probabilities are equal for all behaviors, and we argued that the subject learns precisely this—that action and shock termination are independent. Furthermore, it was argued that the organism would then expect that this will also be true in the future. The difference between the cognitive and the behavioral views is foundational: If an organism is not sensitive to outcomes that occur in the absence of an act (as behaviorism assumed) it cannot determine whether the act does or does not cause the outcome as opposed to merely co-occurring accidentally.

This leaves the issue of how learned act/outcome independence produces later failure to escape in a new environment. Maier and Seligman argued first that part of the motivation to escape is produced by the expectation that action will produce relief, and so *trying* to escape would be undermined by the prior learning. Second, they argued that the learning of non-contingency would *associatively interfere* with learning that there is a contingency in the new situation (proactive interference). We called this collection of ideas the “learned helplessness” hypothesis.

From here the early research on learned helplessness went in four directions. (i) Extension to humans. Experiments in the early 1970s exposed humans to controllable or yoked uncontrollable aversive events such as loud noises or unsolvable anagrams and examined whether the uncontrollable version produced later failure to escape in tasks such as a finger shuttlebox [e.g., (9)]. (ii) Testing the theory in rats. Research in this area quickly switched from dogs to rats. Several other behavioral explanations were offered that did not entail any new principles such as sensitivity to contingency, and we conducted experiments to test these alternative behaviorist views [see review in (7)]. (iii) New sequelae of non-contingent shock other than poor escape/passivity. Inescapable shock turned out to produce a broad range of behavioral changes, not simply poor escape learning. Reduced aggression, reduced food and water intake, disrupted sleep, reduced social interaction, exaggerated fear conditioning, delayed fear extinction, etc. [see review in (10)] all followed inescapable but not equal escapable shock. (iv) Early neuroscience research. Several investigators, most notably H. Anisman and J. Weiss, found brain changes induced by inescapable shock (e.g., norepinephrine depletion in the locus coeruleus) that were involved in producing the sequelae (11, 12). Given the neuroscience tools available in the early 1970s, the implicated processes were, of course, not at a circuit level.

Finally, there were issues that learned helplessness theory had difficulty explaining. (A) Time course. The interference with escape (and later other behavioral changes) following inescapable shock persisted for only 2–3 days [e.g., (1)]. If interference with escape is produced by the prior learning of uncontrollability, why so brief? (B) Active ingredient. The key phenomenon was that uncontrollable shock produced an outcome and exactly equal controllable shock did not. Since the original finding that began this research was that uncontrollable shock produced passivity, we naturally focused on this group and argued that it learned that it has no control and that this learning produced passivity. Our view was that controllable shock fails to produce later interference because it lacks the key ingredient of uncontrollability. However, a possibility that we did not consider is that it is actually control that is learned and this is the active ingredient. By this view exposure to shock—either escapable or inescapable—produces effects, but these effects are inhibited by learning that the shock is controllable [see (13)]. (C) The learned helplessness theory was constructed to explain only later escape failure passivity and it did not readily account for the myriad of other behavioral changes that follow exposure to inescapable shock but are sensitive to controllability. (D) Neuroscience. The early work did not (and could not, given the tools available) make clear how the brain changes found could be modulated by uncontrollability/controllability, nor how such brain changes might lead to the actual behavioral sequelae.

## 3. Considerations for investigating controllability circuity

In reviewing what is known about the neural mechanisms that mediate the impact of behavioral control over stressors, we first discuss criteria for study inclusion. Several experimental paradigms have been used to uncover the neural mechanisms that support uncontrollable/controllable stress effects. Some of these are procedurally quite different from each other, so naturally the phenomena produced by one procedure may be distinct from the phenomena produced by another. This is an important point, as apparent discrepancies in the controllability literature may be due to comparisons being made between different phenomena mediated by different neural processes [see discussion in (14)]. For inclusion, a study must compare the effects of physically identical stressors over which the subjects do and do not have behavioral control over one or more of its characteristics (duration, intensity, etc.).

However, most research that has been presented as relevant to controllability has focused on the mechanisms by which uncontrollable stressors produce their behavioral outcomes. Often a group for which the stressor is controllable is omitted and comparisons only made between subjects exposed to an uncontrollable stressor and subjects not exposed to the stressor. However, in order to demonstrate that some endpoint measure (endocrine, neurochemical, behavioral) is selectively altered by uncontrollability/controllability, the dimension of control must be experimentally manipulated. The inclusion of both an uncontrollable and controllable group (along with a no stress group) allows determination of whether an observed difference is the result of the controllability of the stressor or the stressor *per se*. To see the importance of this, some sequelae of stressors are **not** reduced by having control. For example, inescapable shock leads to a decrease in sucrose preference (15) and daily wheel running activity (16), but equal escapable shock produces those same reductions. Inescapable shock also potentiates defensive responding evoked by innate threat stimuli (17), but again this outcome does not depend on controllability. Similarly, inescapable shock produces robust hypothalamic–pituitary–adrenal (HPA) and autonomic activity, measures often used for assessing the “stressfulness” of an event and for predicting post-stress “susceptible/resilient” phenotypes (18). Both the magnitude and time course of changes in HPA (19–21) and autonomic (22) function occur to the same extent with inescapable and escapable shocks. Many peripheral and central immune responses are driven by adverse events, but these too are independent of control (23, 24). The foregoing suggests that the experience of controllable stress is not simply less “aversive” or “potent” than uncontrollable stress. Rather, *stressor controllability effects arise from select brain circuits and only behaviors that are mediated by those circuits will be affected.*

We have developed a rodent paradigm modeled after Seligman and Maier (2) that manipulates controllability and have used it to develop the neural circuit framework below. The majority of this work has been conducted in male rats, although there is a recent focus on how controllability phenomena differ between the sexes (discussed later in the review). We place rats in small wheel-turn boxes with the rat’s tail extending from the rear of the box with shock electrodes that are fixed to the tail. One group is exposed to a series of tailshocks each of which is terminated when the rat performs a given escape response—ES (turning the wheel with its front paws). Each member of a second group is yoked to a member of ES and is given a series of physically identical tailshocks to those received by the escape group, but these rats have no behavioral control over tailshock termination—IS (turning the wheel has no effect). A third group is placed into the wheel-turn apparatus and does not receive shock—NS. With this arrangement, any observed difference between ES and yoked-IS groups must result from the effect of control, rather than the stressor *per se*, because the shocks are identical for the two groups. Shock is used because the manipulation of control requires a repeatable stressor that can be rapidly initiated and terminated so that ES and IS subjects receive identical physical stimulation. In addition, animals readily learn the controlling/instrumental response for shock termination, with optimal performance achieved within minutes rather than across multiple training sessions. Other aversive events (restraint, social defeat/isolation/crowding, etc.) cannot have their controllability manipulated in any obvious way to ensure that subjects with and without control experience identical physical events.

## 4. Serotonin and the dorsal raphe nucleus

When behavioral outcomes are found to be dependent on controllability, the findings are typically that uncontrollable stress produces behavioral changes that are absent following equivalent controllable stress. We characterize these typical results as either inhibited fight/flight (e.g., reduced aggression) or exaggerated fear (e.g., facilitated fear conditioning). We initially assumed that uncontrollability was the “active element” that initiated neural changes that led to the behavioral changes and that control failed to produce changes because it lacked this critical active element. Thus, the early work focused on identifying the neural systems of uncontrollable stress. Given their diverse nature, we considered ascending neuromodulatory systems the natural starting point given their broad projections to limbic and cortical areas, particularly those that implement the types of behaviors produced by uncontrollable stress (25). At the time, it was known that serotonin (5-HT) cells in the dorsal raphe nucleus (DRN) send topographically organized projections to target structures involved in fight/flight and fear/anxiety-like responses. Specifically, it was known that 5-HT from the DRN released in the amygdala enhances fear, while 5-HT action in the dorsal periaqueductal gray inhibits escape behaviors (26). Additionally, the DRN projects heavily to the dorsal striatum, a structure important for instrumental learning such as escape learning (27). Thus, the known behavioral consequences of uncontrollable stress would occur if uncontrollable stressors activate DRN 5-HT neurons and lead to the release of 5-HT in target structures that are proximate mediators of fight/flight and fear, such as the dorsal periaqueductal gray and amygdala. And, since controllable stressors do not produce those behaviors, they should not activate DRN 5-HT.

This proved to be the case. In the rat DRN approximately 35% neurons are serotonergic and work from the Maier laboratory [see review in (10)] and others implicate 5-HT neurons in the mid to caudal regions of the DRN as critical to the behavioral sequelae of uncontrollable stress. We summarize the most relevant findings: (i) IS, relative to ES, produces an intense activation of 5-HT neurons in mid and caudal DRN as assessed by markers of neural activation in 5-HT labeled cells (e.g., expression of the immediate early gene protein product Fos) (28); (ii) IS, but not ES, increases extracellular 5-HT within the DRN and its projection regions as measured by *in vivo* microdialysis (29); (iii) the potent activation of the DRN by IS results in sensitization of 5-HT neurons for a period of time that equals the duration of behavioral effects, so that excess 5-HT is released in projection regions of the mid/caudal DRN, such as the amygdala and dorsal striatum, in response to input. This period of sensitization by IS (but not ES) is sufficiently profound that even minor simulation, such as exposure to a juvenile rat for 5 min, produces large releases of 5-HT in projection regions of DRN (30); (iii) this large release of 5-HT in projection regions in response to testing conditions is the proximate cause of the behavioral sequelae of IS. We draw this conclusion because the later behavioral effects of IS are blocked by (i) lesion of DRN (31); (ii) inhibition of 5-HT activation at the time of IS (32); (iii) inhibition of DRN 5-HT activation at the time of later behavioral testing (32); (iv) destruction of 5-HT terminals in relevant projection regions (33); and (v) blockade of 5-HT2C receptors during testing (30); (vi) DRN 5-HT activation is not only *necessary* to produce the behavioral sequelae of IS, but it is also *sufficient*. Thus, simply activating DRN 5-HT pharmacologically, in the absence of tailshock, produces the same effects of IS on behavior (34).

The exclusive focus on serotonergic circuitry does not imply that other systems are not involved. Indeed, the work of J. Weiss implicates the noradrenergic locus coeruleus (35) and pharmacological blockade of intra-DRN alpha1 adrenoreceptors prevents IS-induced escape deficits and exaggerated conditioned fear (36). Undoubtedly the behavioral sequelae of IS rely on a complex circuit, and the DRN is just one key structure within that circuit. The DRN is an integrative site toward the efferent end of the circuit, projecting to regions in the brain that are the direct proximate mediators of the behavioral changes. It may be that the locus coeruleus and other structures are critical because they provide excitatory inputs to the DRN that lead to 5-HT activation and its sensitization.

## 5. Behavioral control and the medial prefrontal cortex

By what process do uncontrollable stressors activate the DRN more than equivalent controllable stressors? One possibility is that the process is *intrinsic* to the DRN, with the DRN detecting the presence or absence of the controlling act and after that detection tuning its 5-HT output. If a structure has the capacity to detect whether a stressor is under behavioral control or not, then at a minimum it would need to determine if the outcome (shock termination) was contingent upon the instrumental act (turning a wheel). It seemed unlikely that the relatively small DRN (~30,000 neurons in rat) contained circuitry that would be able to perform this type of analysis and moreover it does not receive the necessary somatosensory and motor inputs. This suggested that the detection of control is *extrinsic* to the DRN. The detection of controllability could be determined by other brain regions that then provide inputs to the DRN responsible for the differential activation of 5-HT cells. Contingency learning is largely a cortical function (37–39) and the DRN receives virtually all its direct cortical input from the medial prefrontal cortex (mPFC) (40–42). Interestingly, converging evidence suggested that the mPFC projections to the DRN provides robust *inhibition* over 5-HT activity. The mPFC-to-DRN pathway originates from layer V pyramidal cells that use the excitatory amino acid, glutamate, as their neurotransmitter. These long-range excitatory projections to DRN preferentially synapse onto DRN GABAergic interneurons that provide a local inhibitory input to 5-HT cells (42). Thus, stimulation of the mPFC and its output to the DRN leads to an inhibition of 5-HT activity (43, 44).

DRN activation during stressors must itself be determined by excitatory inputs, and a number of inputs from the limbic system (lateral habenula/glutamate, locus coeruleus/norepinephrine, bed nucleus of the stria terminalis/corticotropin-releasing hormone, nitric oxide) were known to mediate the effects of uncontrollable stress (36, 45–47). However, none of the inputs were selectively activated during IS; they provided equivalent excitation to DRN 5-HT whether the shock was controllable or uncontrollable [see review in (10)].

**Figure 1:**
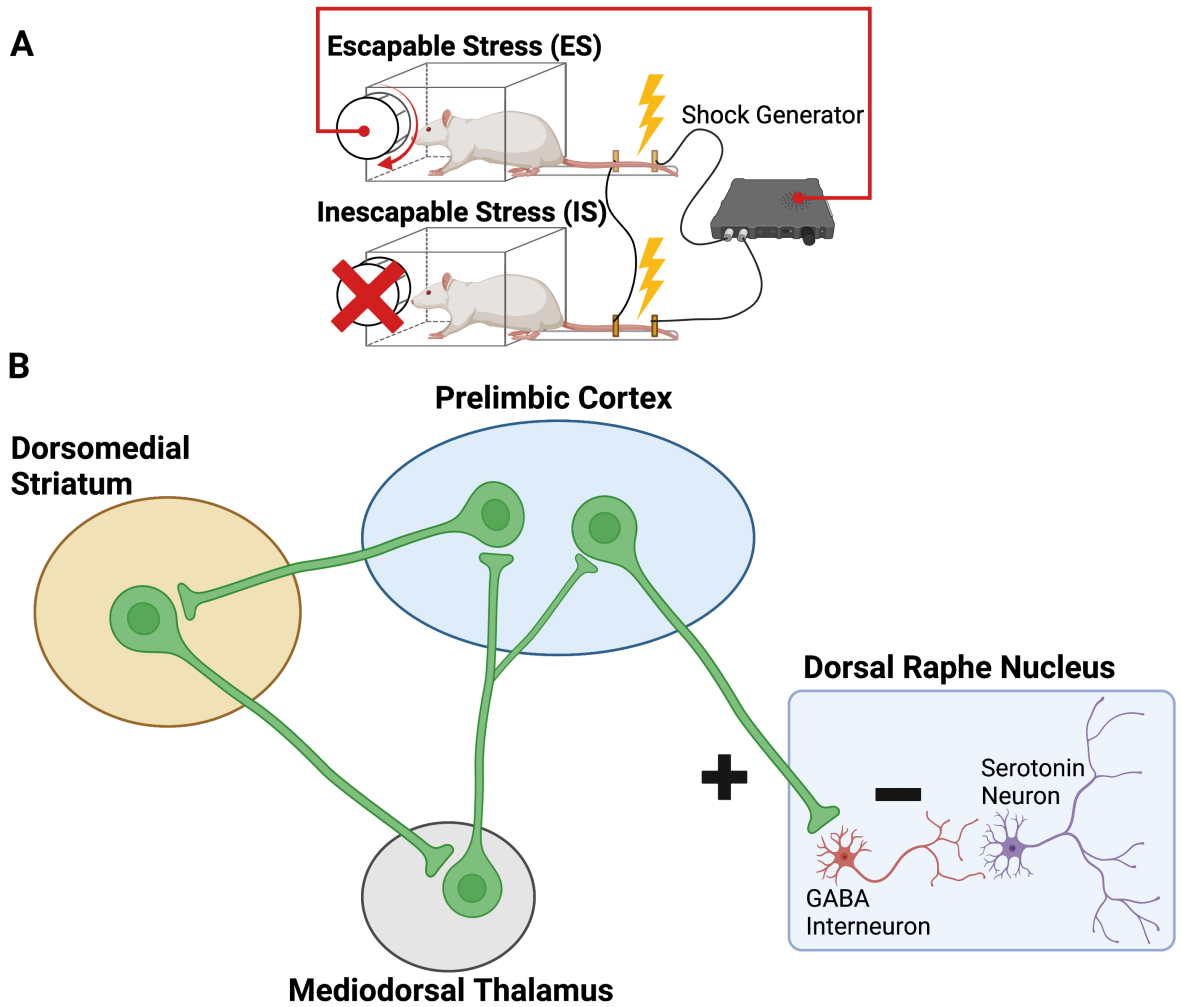
**(A)** Experimental setup for the stressor controllability paradigm. Subjects (typically rats) are assigned to escapable stress (ES), inescapable stress (IS), or no stress (not shown). Subjects receive a series of tailshocks, each of which can be terminated with a wheel-turn response by the ES subject. IS subjects are “yoked” to ES subjects, such that shock is simultaneously terminated for the IS subject when the ES subject achieves the wheel-turn requirement. **(B)** Schematic of the medial prefrontal cortical circuits engaged by escapable stress. “Detection” of the instrumental controlling response is through the goal-directed system, involving the prelimbic cortex (PL), dorsomedial striatum (DMS), and mediodorsal thalamus (MDT). Once control is detected, the PL provides top-down inhibition over the serotonergic dorsal raphe nucleus (DRN) through selective targeting and activation of inhibitory DRN GABA neurons.

If circuits in the mPFC detect controllability, then their inhibitory output to the DRN should only occur when the stressor is *controllable* since ES does not lead to DRN 5-HT activation. The above suggests a framework by which shock-responsive limbic and brainstem structures drive the DRN without regard to shock controllability, with only ES activating an input that inhibits DRN 5-HT activity. Here, behavioral control is viewed as the “active ingredient” in determining the differential activation of the DRN by stressors of differing controllability. The original proposal held that control passively leads to protection because it lacks the active uncontrollability ingredient. Ours is now the exact oppositive of the original: control actively leads to protection (Figure 1).

Our initial attempts directly assessed whether the presence of control activates mPFC neurons that project to the DRN. Baratta et al. (48) delivered a fluorescent retrograde tracer to the mid/caudal regions of the DRN in order to label layer V pyramidal cells of the mPFC. These cells project to the DRN. Rats later received ES, yoked IS, or NS, and then the activation marker Fos was examined in retrogradely labeled mPFC cells. ES, relative to IS, induced Fos expression in DRN-projecting mPFC cells, specifically within the prelimbic cortex (PL), thus demonstrating that behavioral control selectively engages this pathway.

But was this functional? In order to test its functional role, a subsequent experiment inactivated the mPFC during exposure to ES and IS. Inactivation of the mPFC eliminated the DRN 5-HT and behavioral differences between ES and IS. That is, ES rats for whom the mPFC was inactivated now showed the same elevated DRN activity and later behavioral outcomes (impaired shuttlebox escape, exaggerated shock-elicited freezing) as that produced by IS. MPFC inactivation in both IS and NS animals had no effect; the only impact of mPFC silencing was to eliminate the stress-buffering effects of ES (49). Importantly, inactivation did not interfere with the wheel-turn controlling response, and the rats learned to wheel turn at a rate indistinguishable from controls. However, this control no longer blunted the effects of the shocks when the mPFC was silenced. These findings highlight that the mPFC is not necessary to acquire the wheel turning controlling response, rather it most likely is a structure that processes the contingency between the wheel turn and shock termination and subsequently inhibits other structures (such as the DRN) when control is present. Moreover, the data suggest that it is mPFC activation that is necessary for preventing stressor outcomes, not whether the rat has objective control over the stressor.

To further explore this idea, Amat et al. (50) used an opposite strategy to inactivating the mPFC – here the mPFC was pharmacologically *activated* during IS and ES exposure. The results were clear. As is typical, vehicle-treated IS animals later showed poor shuttlebox escape and robust DRN activation. However, **activating the mPFC during IS now led IS to produce the same protection that is observed in ES** rats. Even though IS rats had no control over the shock in the first phase, the DRN response was blunted and they escaped well during later shuttlebox testing.

Further evidence that the protection derived from ES is an active rather than passive process comes from studies of immunization. Initial experience of behavioral control potently blocks the deficits produced by later exposure to uncontrollable shock that occur in a very different environment (**transsituational**) (7, 51). Behavioral control immunizes against the outcomes of both uncontrollable shock *and* other kinds of adversity (**transstimulus**). For example, exposure to ES blocks the behavioral and neurochemical effects of social defeat (52): ES and social defeat were conducted in very different testing environments, on different floors of the building, and by different experimenters. This minimized cues that are shared between the two experiences. Similar to IS, social defeat produces a large increase in DRN 5-HT as well as impaired shuttlebox escape and other behavioral outcomes characteristic of IS. This was prevented if ES was given 1 week before defeat (52).

Thus, ES confers a general protection against subsequent adversity. An additional feature of immunization is that it is **long**-**lasting**. In one example, ES, IS, and NS were given to adolescent rats at 35 days of age. Initial exposure to ES blocked the consequences of IS in adulthood [56 days later; (53)].

How does immunization work? Perhaps an experience with control alters the mPFC-to-DRN pathway in such a way that later uncontrollable events, which normally do not activate this pathway, now do so. Indeed, Fos expression in DRN-projecting mPFC neurons is selectively induced after IS only in rats that received ES 1 week prior (48). Furthermore, both the transsituational and enduring features of immunization require mPFC activity both at the time of initial behavioral control *and* at the time of subsequent IS. That is, silencing the inhibitory pathway at either time prevented immunization (54).

“Plasticity-related” changes to the mPFC are critical for immunization to occur. ES led to the production of plasticity-related proteins in the mPFC (55) and increased excitability of deep layer pyramidal projection neurons of the PL as measured by whole-cell patch-clamp recordings (56). Administration of the protein synthesis inhibitor anisomycin into mPFC after ES had no effect on the immediate protective effects of control (54). The rats learned to wheel turn to end shock, but immunization did not occur. With mPFC protein synthesis prevented there was no stress-buffering impact of prior ES. Other mPFC plasticity prevention, such as blockade of NMDA receptor activity or inhibition of its downstream effector pathway (e.g., mitogen-activated protein kinase, MAPK), also eliminated immunization (55). We conclude that the experience of control induces neuroplasticity in the PL-DRN pathway, and this circuit plasticity causes immunization.

So having control *per se* is not sufficient, rather what is sufficient is whether the mPFC is activated during control. Activating the mPFC during IS was sufficient to immunize against the typical deficits. Amat et al. (50) subsequently asked whether mPFC activation with no shock would also lead to immunization. It did not. However, activation of the mPFC during IS did produce immunization against subsequent IS. That is, mPFC activation in the presence of inescapable shock completely blocked the DRN response to later IS. The DRN responded as if the shock was controllable. Taken together, we conclude that immunization requires (i) conjoint activation of the mPFC in the presence of a stressor and (ii) *de novo* production of plasticity-related protein products. If mPFC activation *during* a stressor proves to be a general mechanism of resilience, future work should be directed at identifying the neural processes engaged by the stressor that must be conjointly present with mPFC activity.

## 6. Relation of the neural circuits to instrumental learning

Clearly, the mPFC projection to the DRN is essential for the stress-buffering actions of behavioral control. The presence of control first needs to be *detected* before the information can be used to inhibit the DRN. “Control” is a similar concept to instrumental learning. In instrumental learning the organism has a voluntary action that produces the desired outcome, i.e., control over the outcome. What are the neural mechanisms of instrumental learning? There are only a relatively small number of studies of the neural mechanisms that mediate aversive instrumental learning, such as escape, avoidance, and punishment [e.g., (57)].

However, there is a large literature investigating the neural mechanisms that mediate appetitive instrumental learning, such as lever pressing for food. Appetitively motivated instrumental responses can be acquired and controlled by two very different systems: Habit learning and goal-directed learning. These two have different associative structures and learning rules [see (58, 59) for reviews]. Any instrumental learning situation involves a voluntary response (R) followed by an outcome (O). This can occur in the presence of specific discrete or contextual cues (S for stimuli). One system, the “goal-directed” (GD) learning system, precisely encodes the contingency between R and O as we defined it above: the difference between the conditional probability of the O in the presence and in the absence of R (38). Thus, if the contingency is weakened or the outcome devalued the organism is less likely to engage in R (60). Here, learning is “contingency-sensitive”: cognitive and flexible as well as independent of context since stimuli are not part of the associative structure encoded by this system (61).

The other system, the habit (H) system, encodes the mere contiguity between S and R and contingency is not a factor. The outcome is not part of the associative structure. Here, learning is non-cognitive, inflexible, and entirely dependent on stimuli present. Neither degrading the R-O contingency nor devaluing the outcome influence the responses in the H system. Each of these systems has benefits and costs. “Goal directed” learning (and “control”) requires determining the causal relationships between acts and outcomes and thereby adjusting action to changes in contingency. However, GD learning is heavy-weight and requires attention and other cognitive resources such as working memory. Habit does not need attention nor other cognitive resources. But habit is inflexible, it is not sensitive to contingency (causality) or changes in outcome value, nor is it deployed when the context changes. These same two systems are present in humans (62) as well as rodents.

These two different learning systems are mediated by different neural circuits. Goal-directed learning in rodents uses the prelimbic subregion (PL) of the mPFC and dorsomedial striatum (DMS, caudate in humans) as well as projections between them (63). More recently, the mediodorsal thalamus (MDT) and its glutamatergic innervation of the PL has also been shown to be important (64). Habit learning, on the other hand is mediated by the dorsolateral striatum (DLS, and putamen in humans) and the sensorimotor cortex (65).

Typically, learning in a new situation first engages the GD system, with behavior shifting to Habit with extended training as it becomes “automatic” (66). However, the shift to Habit can occur quite early in learning and learning can even use the Habit system from the start (67). Furthermore, recent stressors bias both rodents (68) and humans (69) toward reliance on Habit.

Little work has examined whether these instrumental systems are involved in aversively motivated learning. Naturally, since instrumental control and GD learning are similar concepts and both involve the PL, it would follow that instrumental aversive learning might be supported by a similar GD corticostriatal circuitry. Data reviewed above have already indicated involvement of the PL, and Amat et al. (70) showed that ES does preferentially induce Fos in the DMS but not DLS, thus showing that both PL and DMS are activated by ES, as in appetitive instrumental GD learning. Importantly, blockade of NMDA receptors in the DMS during ES prevented the behavioral protection afforded by control and eliminated the differential DRN 5-HT response between ES and IS. In ES subjects, the DRN responded to shock as if it was uncontrollable. In contrast, NMDA receptor blockade in the DLS (part of the Habit system) had no impact on ES protection. Neither of these manipulations interfered with wheel-turn escape performance since instrumental control can be accomplished by either the GD or H system. These data suggested that for behavioral control to produce resilience the controlling action must be acquired and maintained by the GD system.

If true, how is this contingency information then communicated to the PL neurons that inhibit the DRN? The GD system is actually a loop that projects back to the PL. As noted above the GD system involves not only pathways between PL and DMS, but also between PL and MDT. Importantly, the MDT sends glutamatergic projections back to the originating PL structure. We have found that PL-neurons that project to DMS and DRN are separate PL neurons, but are intermixed in PL layer V. Recent evidence demonstrates that individual projecting MDT neurons to the PL innervate a very large number of PL layer V neurons (71), the region where PL-to-DRN neurons are located, suggesting that the very same MDT neurons that participate in contingency detection may also make synaptic contact with PL neurons that regulate the DRN.

If this framework is correct, then activation of the contingency detection circuit should activate the PL cells that use this information to inhibit DRN 5-HT neurons. To begin to explore this possibility, chemogenetic activation of the MDT induces Fos protein in the PL-to-DRN pathway, suggesting a functional link between the circuits involved in the detection and use of control information (72).

## 7. Controllability effects in females

Until recently, virtually all the controllability work had been conducted in male rats. It was completely unknown whether behavioral control phenomena and the neural mechanisms responsible for its operation were present in females. Stress-linked disorders such as major depression, generalized anxiety, and posttraumatic stress disorder have a higher incidence in women than men (73, 74), so an understanding of how coping processes differ between the sexes may be of clinical interest. Surprisingly, we have found that the presence of control in female rats does not blunt the impact of the stressor, nor does it produce behavioral immunization. ES produces the same behavioral outcomes as IS—exaggerated fear, social avoidance, and impaired shuttlebox escape. Moreover, ES in females does not constrain the DRN 5-HT response to shock, and it does not selectively engage PL neurons that project to the DRN as it does in male rats (75, 76).

The lack of a mitigating effect by control is not due to a failure in learning the instrumental wheel-turning controlling response. Females perform the escape response with the same efficacy as males. Recall that instrumental responses can be accomplished with two different learning systems. Protection in males only occurs if the controlling response is acquired with the corticostriatal GD system. One possibility for the lack of protection in females is that the instrumental escape response is supported by different circuitry, namely the prefrontal-independent Habit system. McNulty et al. (77) examined this issue by first quantifying Fos expression in the DMS (GD system) and DLS (Habit system) following ES or IS in females. The Habit system rather than the GD system was preferentially activated during the ES experience in females, the exact opposite pattern previously observed in males. Inactivation of the habit system would bias female ES subjects to acquire the control with the GD system, and indeed, the shift to the GD system led to protection by ES in females. Once again, the implication is that the exercise of behavioral control at a behavioral level is not the critical factor in determining protection, rather it depends upon the circuitry that is recruited during its behavioral performance.

Next, we explored why females might preferentially engage the Habit system during wheel-turning escape. Our starting point took into consideration work from the Arnsten laboratory that clearly shows that excessive levels of catecholamines in the prefrontal cortex, such as those produced by stressors or drugs of abuse, can interfere with mPFC-dependent cognitive functions (78, 79). Notably, persistent high levels of norepinephrine (NE) acting at alpha1-adrenergic receptors, and dopamine (DA) acting at D1 receptors, can impair prefrontal functioning while simultaneously strengthening habit formation and affective responding in humans or rats (80). Sex differences in basal levels and/or stress-evoked release of catecholamines have been reported, with levels generally increased in female compared to male rats (81, 82). Using *in vivo* microdialysis, we monitored in both sexes the extracellular levels of PL DA and NE during wheel-turning escape (77). ES in both males and females led to a robust increase in NE in the PL that remained elevated throughout the entire shock session. The pattern of DA was quite different. In males, ES produced only a transient increase in DA that rapidly returned to basal levels before the end of the ES session. In contrast, the DA increase in females was prolonged and persisted throughout the ES session as well as thereafter. Thus, our data hinted at the possibility that the male/female difference in the beneficial effects of control is due to a greater DA response in the PL during ES in females, thereby biasing toward the use of the Habit system. To address this hypothesis, we microinjected either a D1 antagonist or vehicle into the PL prior to ES. Blockade of PL D1 receptors shifted the proportion of activity-dependent Fos expression from the DLS Habit to the GD DMS system and importantly, now produced protection by ES in females (77). The foregoing represents a sex-selective mechanism in which elevated mPFC DA influences which striatal instrumental system is used for coping with a stressor, and by extension, determines whether coping experiences will translate into resilience.

Sex differences in habit formation extends much more broadly. In the appetitive domain, the type of reinforcement schedule for rats is an important determinant in whether behavior is goal-directed or habitual (83). Ratio schedules are more likely to maintain goal-directed responding because the animal experiences a correlation between its rate of responding and the rate of reward. Interval schedules, in contrast, promote the use of the habit system since the response-reward correlation is degraded. Schoenberg et al. (84) demonstrated that, when trained on a variable-interval schedule of sucrose reinforcement, habit-based performance is accelerated in female rats compared to males. The wheel-turn escape task is also interval-like, and future work should address whether schedules of reinforcement that are more ratio-like would bias instrumental controlling responses in females toward the prefrontal-dependent GD system.

While parallels remain unknown for humans and rats here, we would encourage human investigations of these processes to always looks for sex differences.

## 8. Circuit predictions

These circuits provide insight into several helplessness phenomena that at the time of their initial discovery lacked an explanation. Notably, IS produces (i) a wide variety of outcomes that cut across behavioral dimensions and (ii) persist for only a limited period [2–3 days, (1)]. As reviewed above, IS sequelae are caused by intense activation and subsequent sensitization of DRN 5-HT neurons. The mechanisms for DRN sensitization involve 5HT_1A_ autoreceptors that during activation, inhibit 5-HT cell firing and release. IS, but not ES, leads to prolonged high levels of 5-HT within the DRN which desensitizes 5HT_1A_ receptors. Therefore, only IS subjects exhibit exaggerated 5-HT release in the DRN’s diverse projection regions, the proximate mediators of the behaviors. Thus, the DRN 5HT_1A_ receptor is a key molecular switch in the circuitry determining controllability effects, but its desensitization only lasts for a period of a few days (85), thereby explaining why IS effects are transitory. The widespread projections of DRN to amygdala, periaqueductal gray, etc., explain the widespread impact of IS on behavior.

Importantly, the circuitry predicts that only actions that are modulated by ES- specific circuits will be sensitive to the dimension of control. If the mPFC detects the presence of control and then uses that information to inhibit DRN activity, then perhaps other target structures of the mPFC are also blunted by the experience of control. For instance, activation of a subregion of mPFC, the infralimbic cortex (IL), inhibits fear through its projections to the amygdala, a critical hub for fear (86, 87). Consistent with this, an experience with behavioral control potently inhibits conditioned fear responding, accelerates fear extinction, and, importantly, prevents the spontaneous recovery of fear after extinction (17). Silencing the IL prevents the fear-buffering effects of control (88). As the circuitry would predict, prior ES does not inhibit behaviors that the mPFC does not regulate, such as freezing to a predator odor which is not regulated by the amygdala (17).

We have also pursued new predictions based on control selectively engaging the corticostriatal GD system. The MDT, PL, and their connectivity, substrates of GD learning, are also involved in social dominance. An animal’s dominance status is partly determined by its history of winning in competitive social encounters (89, 90). The tube and warm spot tests are commonly used to assess winning. In the tube test, competitors start at opposite ends of a long clear tube. Winning consists of one subject (“winner”) pushing the other (“loser) out of the tube. In the warm spot test 3 or 4 animals continually compete for single occupancy of a warm spot on a cold cage floor. In both cases, an initial win increases the probability of winning in future competitions (“winner effect”). The MDT and PL neurons of winners are activated during pushing behaviors and repeated winning leads to changes in synaptic strength, including layer V pyramidal output neurons in the PL (90, 91). Manipulations that inhibit or activate the MDT output to the PL resulted in an increase or decrease in social rank, respectively (90).

The authors noted that PL activation most likely did not induce winning by increasing aggression, but rather by initiating and maintaining more effortful behavior. The experience of winning changes the level of persistence/effort that an animal exerts in future competition, and PL activity is required for this to occur. Maier and Seligman (2) reported in the original learned helplessness study that ES subjects persist in their responding during exposure to subsequent IS (increased number of unreinforced panel presses). Prior ES did not merely immunize against the IS deficit, rather it produced responding that was significantly greater than subjects that previously received no stress at all. Control might lead animals to respond more actively toward subsequent adversity, similar to that produced by winning. Perhaps in winning the subject learns that there is a contingency between its effortful behavior (pushing the other competitors out of the tube or off the warm spot) and a positive outcome, and GD circuitry, such as the MDT and PL, are necessary.

For our initial studies we selected the warm spot test (92). If behavioral control and winning strengthen and are regulated by the same PL circuits, then the experience of ES should facilitate later winning on to the warm spot. Rats received ES, yoked IS, or NS control treatment followed by dominance testing 1 week later. Indeed, prior ES potently increased the number of push-backs and occupation time during the warm spot test. IS and No Stress did not differ in their occupancy times. Thus, ES did not merely buffer against IS reducing dominance, it actively produced its own extra effort. To test whether PL activation by ES is critical, the PL was in inhibited during stress. Now, ES no longer facilitated winning even though the wheel-turn response was performed with the same efficacy (92). Thus, control does not merely blunt the impact of adverse events but also facilitates other processes that involve the neurons of the goal directed learning circuit.

Taken together, the neurocircuitry data upend the original thinking that uncontrollability is the active ingredient in producing helplessness. Rather, control, learning that shock termination is dependent on the wheel-turn response is the critical factor. Control selectively engages mPFC-mediated top-down inhibition of the DRN, thereby preventing the debilitating outcomes. The protection afforded by control transfers to new aversive situations, including those that are uncontrollable and those that occur in a novel environment. This resilience only occurs when the instrumental response is learned with structures that support goal-directed, but not habit, learning (70, 77).

## 9. Translating the neuroscience of control to humans: implications for psychotherapy and prevention

There is a developing literature on the neural architecture of control and helplessness in humans. This work is well represented in this volume, and so we will make no attempt to review it here or to evaluate the consistency of the findings with our animal work. The animal neural work also has implications for brain stimulation techniques as therapeutic agents, but discussion is beyond the scope of this paper. Instead, we will concentrate on implications for psychological therapy and prevention.

Learned helplessness is a promising laboratory model of naturally occurring unipolar depression as well as other pathologies in humans (3, 93). The therapeutic emphasis derived from learned helplessness has been on how to undo the debilitating effects of uncontrollability. The rodent neural circuitry evidence above, however, strongly suggests a shift away from undoing the effects of uncontrollability toward **bolstering the beneficial effects of controllability**. This circuitry work built on the demonstrated existence of two very different types of learning circuits uncovered in the study of appetitive instrumental learning: a habit circuit (H) and a goal-directed circuit (GD). The habit system is automatic and, by definition, it is insensitive to changes in external contingencies or the value of the outcome. The goal-directed system in contrast is flexible and it tracks changes in the external contingencies and values of outcomes, adjusting behavior accordingly.

We found that the goal-directed circuits are also used to detect control over aversive events, and this points toward what more effective therapy should be.^1^ The findings reported above show that experience with escapable shock (mastery) only obviates helplessness when it is learned by the GD circuitry, not when it is learned through the non-cognitive habit circuitry. The GD circuitry is cognitive in that it tracks changes in external contingencies and outcome values and adjusts accordingly. This points to the importance of using such cognitions in therapy. So, for therapy to be effective, it should draw upon the goal-directed circuitry not upon the habit circuit.

This bears on a major dispute in therapy. Should the therapist instill habits or cognitions? In the traditional behavioral therapy called “systematic desensitization” for a specific cat phobia, for example, the therapist instills a habit that antagonizes the phobia: the habit of relaxing the whole body to antagonize bodily fear in the presence of the cat. Whereas in cognitive therapy, the therapist teaches the patient to dispute the automatic thoughts (read “mental habits”) that cats are dangerous. The patient disputes the exaggerated cognitions of danger that cause the phobia.

This dispute is at the very heart of the difference between behavior therapy and cognitive therapy. Before behavior therapy and cognitive therapy joined forces in the late 1970s to become “cognitive-behavioral therapy” (Seligman was present at the table), these were two separable and competing endeavors. They joined forces in common opposition to psychoanalysis and humanistic psychology. But this did not paper over the major difference between cognitive and behavioral approaches. For the behavior therapist, coming out of the Hull-Skinner tradition, therapy centered on beneficial habit formation. For the cognitive therapist, coming out of the new cognitive psychology (96, 97), therapy was about arming the “client” with the tools for cognitively disputing the underlying mental habits that caused the problem. Indeed, there is evidence in the animal literature that forcing attention onto a habitual response shifts control of that response to the goal-directed system (61).

Consider depression, for example. The client is rejected by his fiancée. He thinks “I’m unlovable, I’m a loser.” These are automatic thoughts, a pernicious mental state which is accurately called a habit because it is not sensitive to changing contingencies or value of the outcome (“maybe she is not worth it”). The cognitive therapist assists him in disputing this habit with evidence about his successful relations in the past and his successes at work. The client exposes the false automatic thoughts to the light of day, examines them, disputes them, and so abandons them. The behavior therapist, in contrast, ignores the cognitions and focuses on the client’s maladaptive habits, such as avoiding new relationships. The client is encouraged to strike up conversations with attractive strangers and to date anew. The circuitry above suggests that cognitive therapy should be superior, and this indeed is a treatment of choice rather than behavior therapy (3, 98).

The parallel lesson arises for prevention. Seligman and Maier (2) found that prior experience with control over an aversive event “immunized” against helplessness. Organisms that first learned to control shock did not become passive when later exposed to inescapable shock. Recall that ES, IS, and NS were given to adolescent rats at postnatal day 35 (53). Initial exposure to ES blocked the behavioral and neurochemical consequences of IS in adulthood (56 days later). The neural circuitry data, however, shows that this immunization only works if learning about the escapable shock goes through the cognitive goal-directed system.

Parents, teachers, athletic coaches, and purveyors of prevention programs are invested in finding the right early experience to prevent adult problems, such as depression and anxiety. Should the early prevention aim toward creating better habits or should it aim toward better thinking—as the rat data suggest?

There is surely some place for teaching automatic inflexible habits to young children. Survival dictates that not stepping off a curb should become an inflexible habit and parents routinely use punishment to instill this habit. On the other hand, instilling better thinking is in order when the contingencies are less pressing and more complex. When a child comes home with a bad grade what habit should be instilled? No single habit comes to mind. Rather there are several possible contingencies which might have caused the bad grade: e.g., lack of effort, lack of talent, or unfair tests. The child needs to be able to analyze what went wrong (the contingency) and act in accordance. Similarly, when a child falls off her skateboard, the parent should not instill the habit, “do not skateboard,” rather the child needs to analyze which contingency went wrong and so adjust her skateboarding technique.

Prevention and therapy, we conclude, in view of the neural circuitry, should be aimed at better thinking, not better habits.

Translating the rodent circuitry to human therapy and prevention depends on the plausibility of “cognition” in the goal-directed system and the lack of such “cognition” in the habit system. To make our speculative translation more intuitive, consider stopping at a red light while driving. With minimal experience, this becomes an automatic habit: red light, slam on the brake. By contrast, consider passing a moving car. Passing maneuvers are exquisitely sensitive to change. They are not habits. You must take into account several contingencies: your speed versus the others’ speed, the distance between the two of you, your acceleration power, the likelihood that the other will swerve toward you, and the likelihood of impediments in the passing lane. This is a complex set of contingency and outcome value calculations. Importantly, however, these calculations are cognitive, but they do not use conscious verbal thought, a faculty rats do not have. In fact, verbal consciousness of these contingencies does not aid and may even impede passing. Thus, our translation is not dependent on the presence of conscious awareness, only on a cognitive faculty that rats and humans share: calculating the changing contingencies and outcome values and adjusting action accordingly.

However, there is a complication. Activation of the GD circuitry only produces immunization if this happens during the experience of an aversive event. Simply activating the GD circuits by themselves without adversity did not produce immunization. Both are needed to activate the pathways from the PL to structures such as the DRN, thereby inducing plasticity in these pathways so that they are later activated even by uncontrollable events, thereby producing protection. Translation would therefore suggest that the prevention strategies discussed above would be especially useful in the context of unpleasant circumstances. Indeed, if a person experiences only positive events, immunization against adversity may not be possible. This combination is not a contraindication for therapy since effective therapy is usually done in the context of adversity.

## 10. Translating the neuroscience of control to humans: implications for biological therapies

The circuitry that we have uncovered in rodents may help to understand the therapeutic efficacy as well as the complexity of several biological treatments. To set the stage, here is a summary of the rat circuitry: Control blunts the impact of stressors experienced during control because it activates glutamatergic PL pyramidal neurons that project to stress-responsive limbic and brainstem structures such as the DRN and leads to immunization because it induces plasticity in these pathways. Control activates the PL-DRN pathway because it engages the GD learning circuitry while a stressor is present. However, any other manipulation that activates PL-DRN might also be therapeutic. Here we briefly consider three.

1) Direct brain stimulation. The PL neurons that project to the DRN are in layer V, and only a small percentage of these output neurons (perhaps 5%) project to the DRN. Thus, it would be difficult to target such a specific population of cells in humans. There is also uncertainty regarding which area of the human prefrontal cortex is homologous to the rat PL (99), but it has been suggested that this is Brodmann’s Area 32, comprising the rostral division of the anterior cingulate cortex (100, 101). Stimulation of a focal cortical area that contains these DRN-projecting cells might be therapeutic, however stimulation would also simultaneously impact cells that participate in circuits subserving other functions or even functions that interfere with output to the DRN. Even if human circuit tools were capable of selectively stimulating PL output neurons, only a small fraction project to the DRN (41). Thus, identifying some biological property (e.g., transcript, protein) that is unique to the PL cells that provide output to the DRN is a critical first step that could make the circuit usable for intervention by taking advantage of that property. Alternatively, an intermediate approach would be one that paired stimulation with a psychosocial procedure that engage the relevant circuits. There is some early evidence that non-invasive stimulation is more efficacious when the target circuits are labile (102).
2) Ketamine. The partial NMDA antagonist ketamine produces rapid anti-depressant effects that persist for several weeks after a single administration (103). Although ketamine has multiple effects (104, 105), it activates glutamatergic mPFC neurons, including PL neurons that project to the DRN (106). Indeed, we find that ketamine blocks the DRN activation produced by IS (106, 107). Interestingly, we have found that ketamine also induces plasticity in the PL-DRN pathway. This would suggest that ketamine should produce resilience to future uncontrollable stressors, and this proved to be the case (106, 108). A single dose of ketamine blocked the behavioral effects of IS occurring 1 month later, as well as DRN activation. Whether ketamine might produce prevention in humans is an active area of investigation (109).
3) Psilocybin. Psilocybin has rapid and persistent therapeutic effects (110). Psilocybin targets primarily the 5-HT2A receptor (111, 112), and interestingly, these receptors are densely expressed on the soma and dendrites of PL glutamatergic layer V neurons, although the connectivity of these cells has yet to be known (113). 5-HT2A receptors are excitatory, and thus psilocybin activates the output neurons of the rodent mPFC (113). Thus, it would be expected that psilocybin would activate PL neurons that project to the DRN and other stress-responsive lower structures. Interestingly, a single dose of psilocybin can induce plasticity in layer V mPFC neurons (114), although whether it does so in the PL-DRN pathway is not known. It is possible that this circuitry could account for psilocybin’s beneficial effects.

## 11. Conclusion

Our work began with a laboratory curiosity—subjects exposed to Pavlovian conditioning with electric shock UCSs, later failed to learn to escape shock in a different situation where escape was possible. We quickly focused on the learning of uncontrollability as cause, but research of the last 20 years suggests instead that the processing of the presence, not the absence, of control is the active ingredient. When control is processed by goal-directed learning circuitry and an aversive event is present, descending pathways are activated from the medial prefrontal cortex, that inhibit stress-responsive limbic and brainstem structures such as the dorsal raphe nucleus, leading to resilience in the face of adversity. In addition, these descending pathways undergo persistent plasticity-related changes, thereby producing long-lasting resilience to a broad range of challenges. Control processing by habit circuitry does not activate the resilience-producing descending circuitry. This suggests that therapy and prevention for depression in humans might better rely on building cognitions of mastery, rather than building habits. We look forward to new approaches to intervention that will find other manipulations—behavioral, pharmacological, and physiological—that can harness this circuitry.

## Author contributions

MB, MS, and SM wrote and revised the manuscript. All authors contributed to the article and approved the submitted version.

## Funding

The research described here, and the preparation of this manuscript was supported by NIH Grants R01 MH050479 (SM) and R21 MH116353 (MB).

## Conflict of interest

The authors declare that the research was conducted in the absence of any commercial or financial relationships that could be construed as a potential conflict of interest.

## Publisher’s note

All claims expressed in this article are solely those of the authors and do not necessarily represent those of their affiliated organizations, or those of the publisher, the editors and the reviewers. Any product that may be evaluated in this article, or claim that may be made by its manufacturer, is not guaranteed or endorsed by the publisher.

## Footnotes

^1^It should be noted that these same circuits have been implicated in drug addiction, with addictive behavior being driven by the habit system, and similar conclusions to those below have been drawn for implications for treating addiction (94, 95). However, discussion of addiction is beyond the scope of this paper.
